# A simple way for targeted delivery of an antibiotic: *In vitro* evaluation of a nanoclay-based composite

**DOI:** 10.1371/journal.pone.0187879

**Published:** 2017-11-17

**Authors:** Leslie Valdés, Irela Pérez, Louis Charles de Ménorval, Ernesto Altshuler, Jon Otto Fossum, Aramis Rivera

**Affiliations:** 1 Department of Pharmacy, Institute of Pharmacy and Food (IFAL), University of Havana, Havana, Cuba; 2 Institut Charles Gerhardt Montpellier, Equipe Agregats, Interface, et Materiaux pour l'Energie (AIME), Université Montpellier 2, Montpellier, France; 3 Group of Complex Systems and Statistical Physics, Physics Faculty, University of Havana, Havana, Cuba; 4 Department of Physics, Norwegian University of Science and Technology (NTNU), Trondheim, Norway; 5 Zeolites Engineering Laboratory, Institute of Materials Science and Technology (IMRE), University of Havana, Havana, Cuba; Institute of Materials Science, GERMANY

## Abstract

The sodium-modified form of fluorohectorite nanoclay (NaFh) is introduced as a potential drug carrier, demonstrating its ability for the controlled release of the broad-spectrum antibiotic Ciprofloxacin through *in vitro* tests. The new clay-drug composite is designed to target the local infections in the large intestine, where it delivers most of the incorporated drug thanks to its pH-sensitive behavior. The composite has been conceived to avoid the use of coating technology and to decrease the side-effects commonly associated to the burst-release of the ciprofloxacin at the stomach level. NaFh was obtained from lithium-fluorohectorite by ion exchange, and its lack of toxicity was demonstrated by *in vivo* studies. Ciprofloxacin hydrochloride (Cipro) was encapsulated into the clay at different values of the pH, drug initial concentration, temperature and time. Systematic studies by X-ray diffraction (XRD), infrared and visible spectrophotometry (FT-IR and UV-vis), and thermal analysis (TGA) indicated that the NaFh host exhibits a high encapsulation efficiency for Cipro, which reaches a 90% of the initial Cipro in solution at 65 ^o^C, with initial concentration of drug in solution of 1.36 x 10^−2^ mol L^-1^ at acid pH. XRD revealed that a true intercalation of Cipro takes place between clay layers. TG showed an increased thermal stability of the drug when intercalated into the clay, as compared to the “free” Cipro. IR suggested a strong clay-Cipro interaction via ketone group, as well as the establishment of hydrogen bonds between the two materials. *In vitro* drug release tests revealed that NaFh is a potentially efficient carrier to deliver Cipro in the large intestine, where the release process is mediated by more than just one mechanism.

## Introduction

Clays and clay minerals are used in the pharmaceutical industry as excipients or active pharmaceutical ingredients [1–3]. The structure of these materials determines their chemical and physical properties―high specific area, sorptive and ionic exchange capacities, rheological properties, chemically inertness and low or null toxicity for the patient [4]―for which they are used in pharmaceutical formulations. Some authors report a decrease in the bioavailability of drugs due to the co-administration of clays in the formulation [5–7]. However, the joint administration of drugs and clays has widely accepted advantages from the biopharmaceutical, pharmacological and chemical points of view [5–8]. It results in improved drug solubility and/or modification of its release profile, prevention or reduction of side effects, as well as an increased stability of the drug [9].

In the smectites group of clays ―hectorite, saponite and montmorillonite―the last two have been the most commonly used for pharmaceutical purposes because of their higher cation exchange capacity as compared to other pharmaceutical silicates (such as talc, kaolin and fibrous clay minerals) [5, 7, 10]. In the montmorillonite the layer charge originates from the substitution of dioctahedral Al^3+^ by Mg^2+^. For the hectorite, which is also a 2:1 layer silicate, the octahedral charge of 1.2 e^-^ by unit cell (Si_8_O_20_) generates from substitution of Li^+^ for Mg^2+^ in the trioctahedral sheet [4, 11]. In the fluorohectorite, all the OH groups have been replaced by F^-^, and it is characterized by a layered structure with a layer thickness in the nanometer range [11]. The cation substitution in the octahedral layer results in a structural negative charge, which is compensated by exchangeable hydrated cations to balance the charge and allow their stacking [11, 12]. Such particle stacks can swell in the presence of water, which may enter the interlayer space, increasing the distance between layers. The swelling phenomenon is one of the most remarkable features of the smectite clays. It is controled by charge-compensating counter-ions, which could displace the natural cation located in the interlamellar space [13, 14].

Ciprofloxacin is a second generation synthetic chemotherapeutic antibiotic of the fluoroquinolone drug class. Unlike most broad-spectrum antibacterial drugs, ciprofloxacin is effective both after oral or intravenous administration [15–17]. The high rate of absorption at the stomach of some drugs ―as in the case of ciprofloxacin― have demanded dosage forms ensuring a constant *in vivo* drug concentration in the pH conditions of the intestinal lumen over the full dosage period while preventing harmful side-effects and drawbacks [18, 19]. In this sense, colon targeting is naturally of value for the local and topical treatment of inflammatory bowel disease by means of the antibiotics target delivery systems [20]. In addition, it is well known that the extended oral administration of antibiotics can cause gastric-lesions during long periods [21, 22], and a way to reduce it is by using clays which possess important bioadhesive properties, and gastroprotective antacid activity [23]. The resulting intercalation products can solve these problems by improving the drug properties and effectiveness, reducing its toxicity, and prolonging the half-lives in blood. Accordingly, several materials have been developed as drugs hosts [24–28], constituting a challenge for sustained release developers. It is worth noticing the successful use of synthetic and natural clays as drug carriers [8, 29–32]. Our aim is to develop a clay-based preparation for the controlled delivery of ciprofloxacin targeted at infections in the large intestine, able to avoid side effects at the gastric level due to burst release. In addition, the proposed preparation avoids the use if coating technology, which involves technologically demanding processes whose details are not fully understood [33]. To the best of our knowledge, clay-based products with such properties have not been reported before.

In the present work we investigate the use of the sodium modified form of fluorohectorite (NaFh) ―obtained from LiFh through of an ion exchange process― as an efficient drug carrier. In order to support the potential pharmaceutical application of sodium fluorohectorite, *in vivo* acute toxicity assays were performed for the starting fluorohectorite according to standard pharmaceutical requirements. The effect of pH, drug initial concentration, temperature and time as a function of Cipro incorporation into NaFh were evaluated. Furthermore, the raw and composite materials were characterized by means of X-ray powder diffraction (XRD), Fourier transform infrared spectroscopy (FT-IR) and thermal gravimetric analysis (TGA). Ciprofloxacin incorporation efficiency in NaFh, as well as *in vitro* drug release profiles in both simulated gastric (SGF) and intestinal fluids (SIF) were quantified using ultraviolet (UV) spectroscopy.

Finally, we performed *in vitro* tests to evaluate the release of the antibiotic from the composite at pH values typical of different sections of the gastrointestinal track, which demonstrated its potential for the control delivery of ciprofloxacin in the large intestine.

## Materials and methods

### Materials

The raw material used in this work is the synthetic fluorohectorite (LiFh) produced by Corning Inc. (New York). Its chemical composition is M_x_(Mg_6-x_Li_x_)F_4_Si_8_O_20_, where M denotes the exchangeable cations, and ideally, x = 1.2 [11]. From previous work by X-ray diffraction (XRD) and Atomic Absorption Spectrometry (AAS), it has been demonstrated that the LiFh contains about 80% by mass of LiFh clay, and about 20% of Li_2_O·2SiO_2_ impurities [32]. LiFh was submitted to a chemical modification with sodium chloride (NaCl) following the ionic exchange procedure described in [34], in order to obtain its sodium modified form, NaFh. It is known that the lithium ions are commonly used in the treatment of different pathologies [35], so their unjustified or excessive use could cause side effects in the human organism. Thus, the studies carried out in the present work were performed on NaFh samples, considering that the sodium ions ―which would leave from the material in aqueous environment ―are friendlier to the human body. The model drug of choice was Ciprofloxacin in the form of ciprofloxacin hydrochloride (C_17_H_19_ClFN_3_O_3_), pharmaceutical-grade according to Pharmacopoeia [1], which was used as received from the Cuban pharmaceutical industry. All other chemicals used in the study were analytical grade.

### Acute toxicity assay

The study was approved by the institutional Animals Care Committee from Center of study for the Research and Biological Evaluation (CEIEB) from Institute of Pharmacy and Food (IFAL), University of Havana, Cuba. The animals were supplied by the Center of Laboratory Animals (CENPALAB, La Havana, Cuba) with certified health.

Considering the great importance of raw materials in pharmaceutical formulations, toxicological studies were performed on LiFh samples. The aim is to evaluate the safety of the LiFh when it or its modified forms are administrated orally to experimentation animals. The tests were made following the procedure described by the Organization for Economic Co-operation and Development (OECD TG 423), and established by the Center of Study for the Research and Biological Evaluation (CEIEB) from Institute of Pharmacy and Food (IFAL), University of Havana, Cuba [36–38]. The study was carried out in Wistar albino rats, which were supplied by the Center of Laboratory Animals (CENPALAB, La Habana, Cuba) with certified health, and a corporal weight between 175 and 200 g. The quarantine conditions were: room temperature of 20 ± 3°C, relative humidity in the range (30–70) ± 5%, light/darkness cycle of 12/12 h, and acclimatized for five days prior to experimentation. Water and food were supplied “ad libitum” (Standard diets for rodents, CENPALAB).

Before starting the study, all the rats were weighed in order to use the correct dosage according to the weight of the animals. A maximum dose of 2000 mg·Kg^-1^ doses of LiFh clay were given in a volume up to 0.004 L per 200 g of weight. The clay was administrated to a first group of three female rats. The animals were weighted during the days 1, 7 and 14, and possible delayed toxicity signs were evaluated. The observation took place individually for each rat several times during the first day of the study and once a day during the rest of the experiment, which was carried out during two weeks. During the test no deaths were found, thus the same doses were supplied to another group of three rats of the same sex. All procedures had been approved by the institutional Animals Care Committee, which are in accordance with the European Union Guidelines for Animals Experimentation. At the end of each assay, the rats under study were euthanized ‒with an over-dosage of barbiturate‒ in order to examine internal organs, following the principles of the 3Rs. If any alteration was detected on the organs (lungs, heart, spleen, kidneys and stomach) by simple inspection, a sample was submitted to a histopathology study.

### Drug incorporation and pre-formulation of the drug carrier system

To study the effect of different physical parameters (pH, drug concentration, temperature and time) on the adsorption of ciprofloxacin on NaFh, the following general procedure was followed: 0.03 L of aqueous solutions of ciprofloxacin was mixed with 0.3 g of NaFh powder in a glass-covered flask under magnetic agitation. After the interaction, the mixture was centrifuged for 15 min at 300 rpm. The drug contents in the supernatant solutions were determined by means of ultraviolet (UV) spectroscopy relative to calibration curves for the pure Cipro solutions, according to standard procedures [1]. The UV spectra were collected using a Rayleigh UV-2601 spectrophotometer in the wavelength interval 200–400 nm (λ_max_ = 276 nm). The resulting solid composites were dried at 65°C.

At this point, the different parameters mentioned above were evaluated. The influence of the pH on the suspensions (NaFh–ciprofloxacin solution) on drug incorporation into clay was studied in the following ranges: 3.0–3.5, 6.5–7.0 and 10–10.5. The pH values were carefully adjusted by means of concentrated solutions of hydrochloric acid (HCl) and sodium hydroxide (NaOH) concentrated. The suspensions were stirred for 4 h between 65–70°C. The initial drug concentration was 8.15 x 10^-3^mol L^-1^.

Kinetic studies were performed at acid pH in the range of 65–70°C and at 8.15 x 10^−3^ mol L^-1^ of Cipro solution. To evaluate the effect of the ciprofloxacin concentration, the drug–clay suspensions were prepared at different drug initial concentrations (8.15 x 10^−3^ mol L^-1^, 9.5 x 10^−3^, 1.11 x 10^−2^, 1.22 x 10^−2^, 1.36 x 10^−2^, 1.50 x 10^−2^ and 1.63 x 10^−2^ mol L^-1^) at acid pH for 4 h between 65–70°C. The temperature effect was also assessed carrying out the drug incorporation experiments at 30, 45, 55, 65, 75, 80 and 85°C± 3°C at acid pH, 4 h of contact and 1.36 x 10^−2^ mol L^-1^of drug initial concentration.

Each parameter was studied in five different batches and the analysis was replicated three times for each one; the resulting average value was used for data analysis. The maximum difference between the outputs and their average was of 2.72 x 10^−5^ mol, corresponding to a relative uncertainty of approximately 5% in the mass of incorporated drug.

The Cipro uptake by the clay (i.e., adsorbent loading) was calculated as:

$$q_{e}=\frac{{(C}_{o}-C_{f})\times V}{m}$$

where *q*_*e*_ (mol·g^-1^) is the mass of adsorbed Cipro per unit mass of the adsorbent, *C*_*o*_ is the initial concentration of Cipro solution (mol·L^-1^), *C*_*f*_ is the final concentration of Cipro solution (mol·L^-1^), *V* is the volume of solution (L) and m is the mass of the adsorbent (g) used in the experiments.

### Characterization of the solids

Thermogravimetric (TG) analysis of the samples (Cipro, NaFh and NaFh-Cipro) was carried out with the aid of a NETZSCH STA 409 PC/PG thermal analyser, using air at a flow rate of 0.05 L·min^-1^and a heating rate of 10°C/min from 20 to 800°C. The sensitivity of the thermobalance was ±1 μg. A solid sample of about 0.3g was used in each test.

The X-ray diffraction (XRD) patters for the different samples (Cipro, NaFh and NaFh-Cipro composite) were recorder on a Philips Xpert diffractometer, using Cu K*α* radiation (λ = 1.54 Å) at room temperature, operating at a voltage of 45 kV and working current 25 mA. The experiments were done at a scan rate of 1° min^-1^ for a 2θ range spanned from 2 to 40°.

Fourier transform infrared spectra (FT-IR) were collected in the wavenumber interval 400 to 4000 cm^-1^ using a Nicolet AVATAR 330 Fourier–transform IR spectrometer. In order to prepare for FT-IR analysis, the solids before and after the contact with the Cipro were previously dried overnight in an oven at 100°C and 65°C for the native clay, and the Cipro and the NaFh-Cipro composite, respectively. The samples were prepared using KBr pellet with 0.8% inclusion of the material to be analyzed.

### Drug release experiments

Firstly, two separate release experiments were performed on 0.3 g samples of the NaFh-Cipro composite at pH values of 1.2 (simulated gastric fluid without pepsine, i.e., 2 g of NaCl in 1L of HCl 0.1N) and 7.0 (simulated intestinal fluid i.e., 6.8 g of K_2_HPO_4_ in 1L of NaOH 0.2N). Those values correspond to the environments of the stomach and the large intestine, respectively. In order to simulate more realistically the whole passage through the gastrointestinal tract, 0.3 g of composite NaFh-Cipro were first put in contact with a buffer solution at pH = 1.2 during 2 h, as the average gastric emptying time is about 2 h. After that, the suspension was centrifuged, and the re-suspension of the composite took place in a buffer solution at pH = 7.0 and tested for further 4 h. These conditions reproduce the oral drug administration and the subsequent physiological release. 0.30 g of NaFh-Cipro composite (equivalents to 2.53 x 10^−5^ mol of Cipro determined by UV as described before) were put in contact with 0.05 L of dissolution medium with a shaking frequency of 100 rpm at 37 ^o^C, according to the method reported in the Pharmacopoeia for this kind of systems [1]. Aliquots of the solutions (0.002 L) were collected at regular time intervals, and centrifuged. Each time, the same volume was replaced with fresh dissolution medium. The Cipro concentration as a function of time was monitored by mean of UV spectroscopy at λ_max_ = 276 nm. Release studies were performed in triplicate and the analysis was replicated three times for each one. The average values of the release percentage were reported, with a relative uncertainty of a 5%, corresponding to the maximum difference in release percentage between the average and the replicas.

To evaluate the mechanism of Cipro release from the NaFh-Cipro composites, the in vitro release data was fitted by different mathematical models.

## Results and discussion

### In vivo acute toxicity testing

During the test, no dead animals were found. On the contrary, the results indicate that a gain of weight after the administration of LiFh clay for different periods of time took place, which suggest the absence of systematic toxic effects. In addition, no clinical signs of toxicity were detected when the organs and different system related were examined in the animal groups under study. The samples of the selected organs in the autopsies did not show any alteration from the macroscopic point of view. For this reason, the histopathology study did not take place. The LiFh did not produce acute oral toxicity in the experimentation animals using the corresponding assay described in the OECD TG 423 [37] with a dose of 2000 mg per kilogram of corporal weight, classifying the LiFh as “*without classification*” according to the European Union. Therefore, such results support the use of the raw material employed in the present work for medical applications.

### Effect of various chemical and physical factors on drug adsorption onto NaFh

#### Influence of the drug-clay suspension pH, and the initial concentration of the ciprofloxacin solution on its incorporation in clay

**Fig 1:**
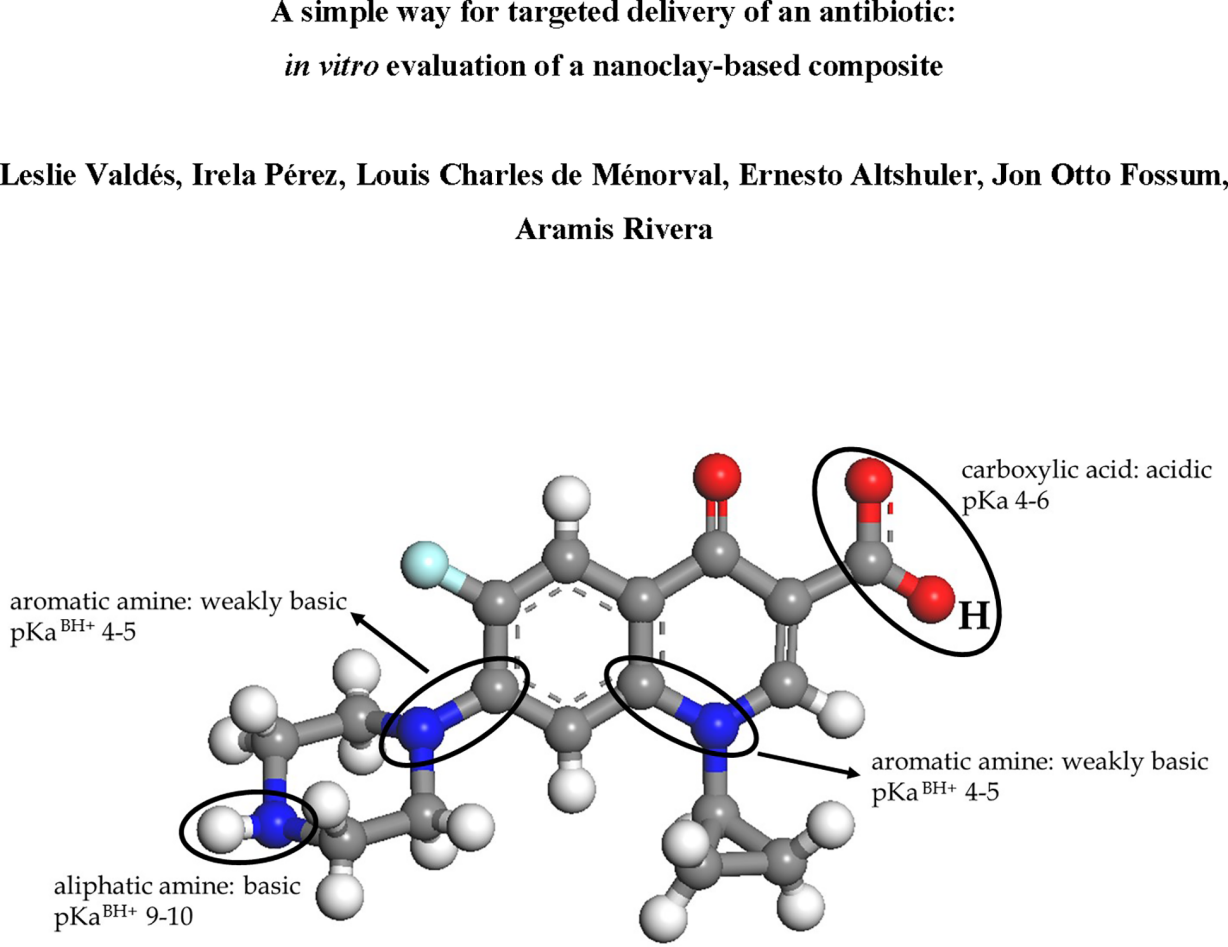
Molecular structure of the ciprofloxacin, and estimated pKa values of the different functional groups—BH^+^ is referred to their degree ionization.

The results regarding effect of pH (3.5–4.0, 6–6.5 and 10–10.5) on the incorporation of Cipro on the clay suggest that it is similar at acid and neutral pH (around 0.80 x 10^−3^ mol g^-1^). However, at basic pH the intercalation of Cipro in clay decreases abruptly, indicating a very poor adsorption of the drug by the clay. Such behavior can be explained based on estimated pKa values [39, 40] for the Cipro molecule (see Fig 1): at acid pH, pH values lower than all the estimated pKa values, Cipro will be protonated by the aliphatic amine group in the piperzine moiety, even on the weakly basic aromatic amine (cationic form of the molecule). At “neutral” pH, all the groups protonable with pKa < 7 (aromatic ammonium ions, carboxylic acid) lose the hydrogen atoms, thus establishing a balance of charges on the molecule. This balance is characteristic of the zwitterionic form of the Cipro. However, at basic pH after passing the second pKa, the proton from the aliphatic amine group is lost, and the anionic form of Cipro is dominant in solution. Hence, at acid and neutral pH where the capture of Cipro is quite high, the interaction drug–clay may takes place between several positively-charged, pinning points of the drug,—*i*.*e*., *via carboxylic acid and aromatic ammonium groups*, *or aliphatic amine group*—and the negative charge of the clays layers. In addition, it should be noted that as the difference in Cipro incorporation on the clay at acid and neutral pH is negligible, a decision was taken to work at acid pH because it is easier from the experimental point of view.

**Fig 2:**
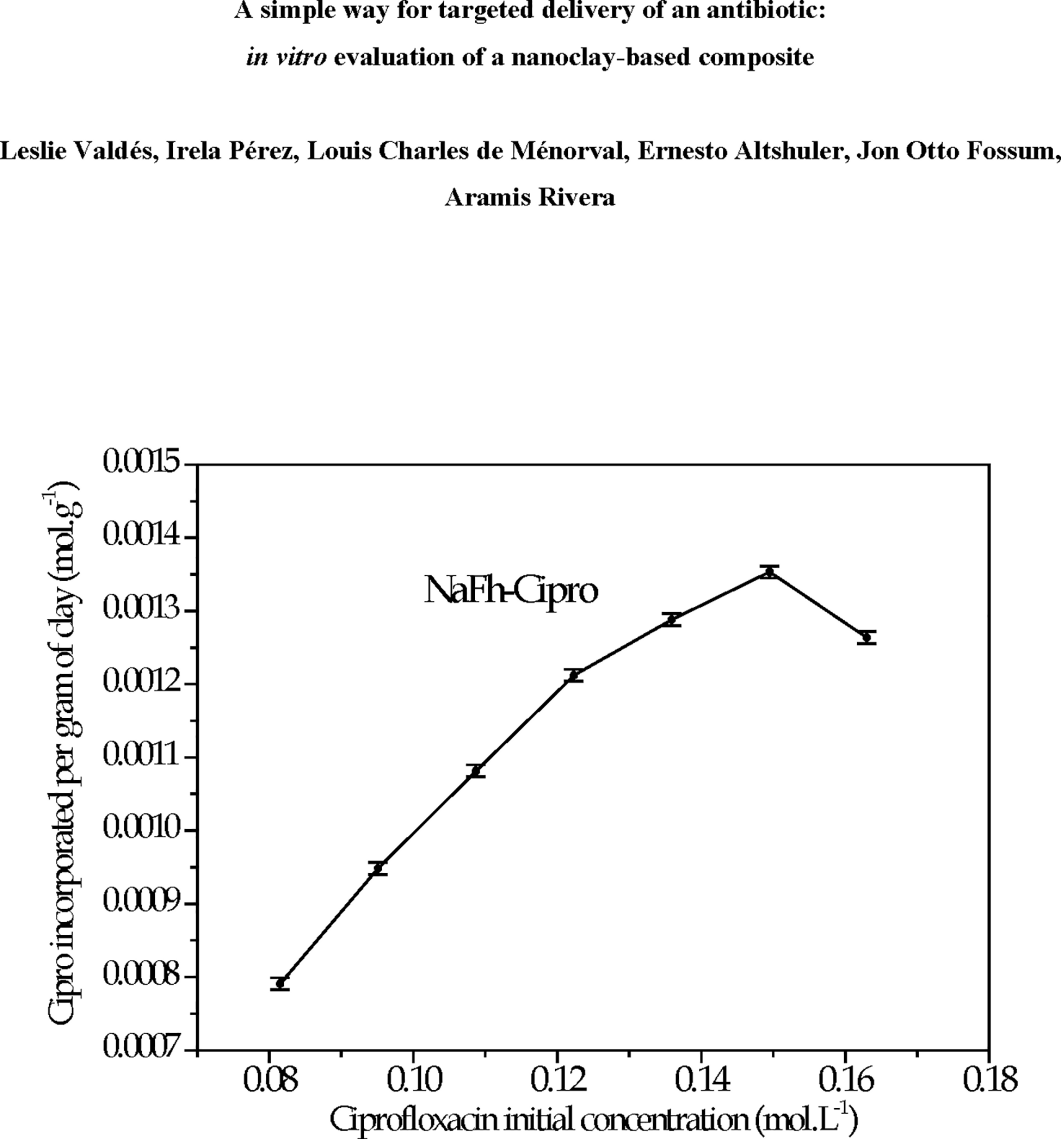
Dependence of the amount of Cipro incorporated into clay with the drug initial concentration.

It was verified that the Cipro intercalation in NaFh is affected by the drug initial concentration (see Fig 2). The Cipro amount incorporated into the clay increases linearly with the drug initial concentration until 1.36 x 10^−2^ mol L^-1^. After that, the drug incorporation efficiency ―Cipro amount adsorbed in the clay *vs* drug mass in initial solution before interaction with the clay― starts to decrease. Based on it, the Cipro initial concentration selected for the following experiments was 1.36 x 10^−2^ mol L^-1^, which corresponds to 1.30 x 10^−3^ mol of ciprofloxacin per gram of NaFh.

#### Temperature effects, and intercalation kinetics

**Fig 3:**
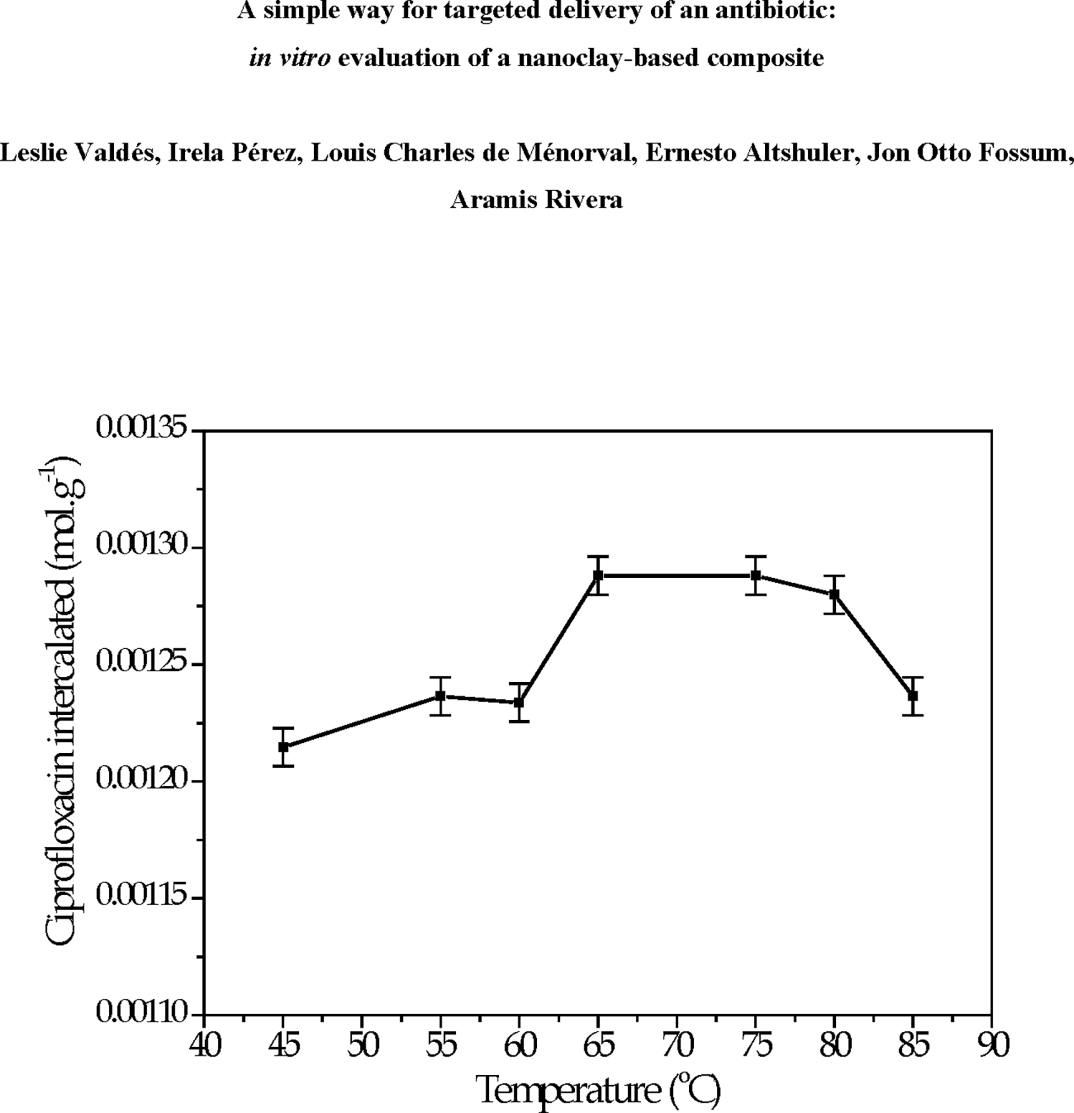
Cipro load onto NaFh from aqueous solution at different temperatures at 1.36 x 10^−2^ mol L^-1^ and equilibration time 6 h.

In previous work, it had been demonstrated that the swelling of the clay under study is temperature dependent. At low temperatures, net attractive interactions between clay particles are dominant, so they remain attached to each other in stacks. However, at higher temperatures it is energetically favorable for the clay to swell due to the entropy that is gained by counterions, which are liberated during swelling [41]. It has a positive influence on its capacity to incorporate chemical species, which is corroborated by the results shown in Fig 3. It is observed that, with increasing temperature the Cipro incorporation becomes bigger, reaching a plateau around 65–75 ^o^C. For higher temperatures, the Cipro amount incorporated decreases. Such behavior is explained based on the studies made by Hansen et al., on a Na-fluorohectorite (NaFh) clay [41], who reported that when the temperature was increased, large changes in the scattering were observed. As temperature increases to 78 ^o^C, swelling occurs as particle stacks delaminate into much smaller stacks or single unit layers and many charge-balancing interlayer cations become free to conduct. Thus, the NaFh particles pass from a passive state at low temperatures to an active, swollen state as the temperature is increased from which no further changes in the scattering takes place, indicating that a stable state has been reached. This swelling transition greatly changes the material properties of the clay. Thus, in the present work an optimal temperature range is expected, where the amount of incorporated Cipro is invariable and stable, i.e., around 65–75 ^o^C. Hence, this temperature range is the one employed in the rest of the studies described from now on.

**Fig 4:**
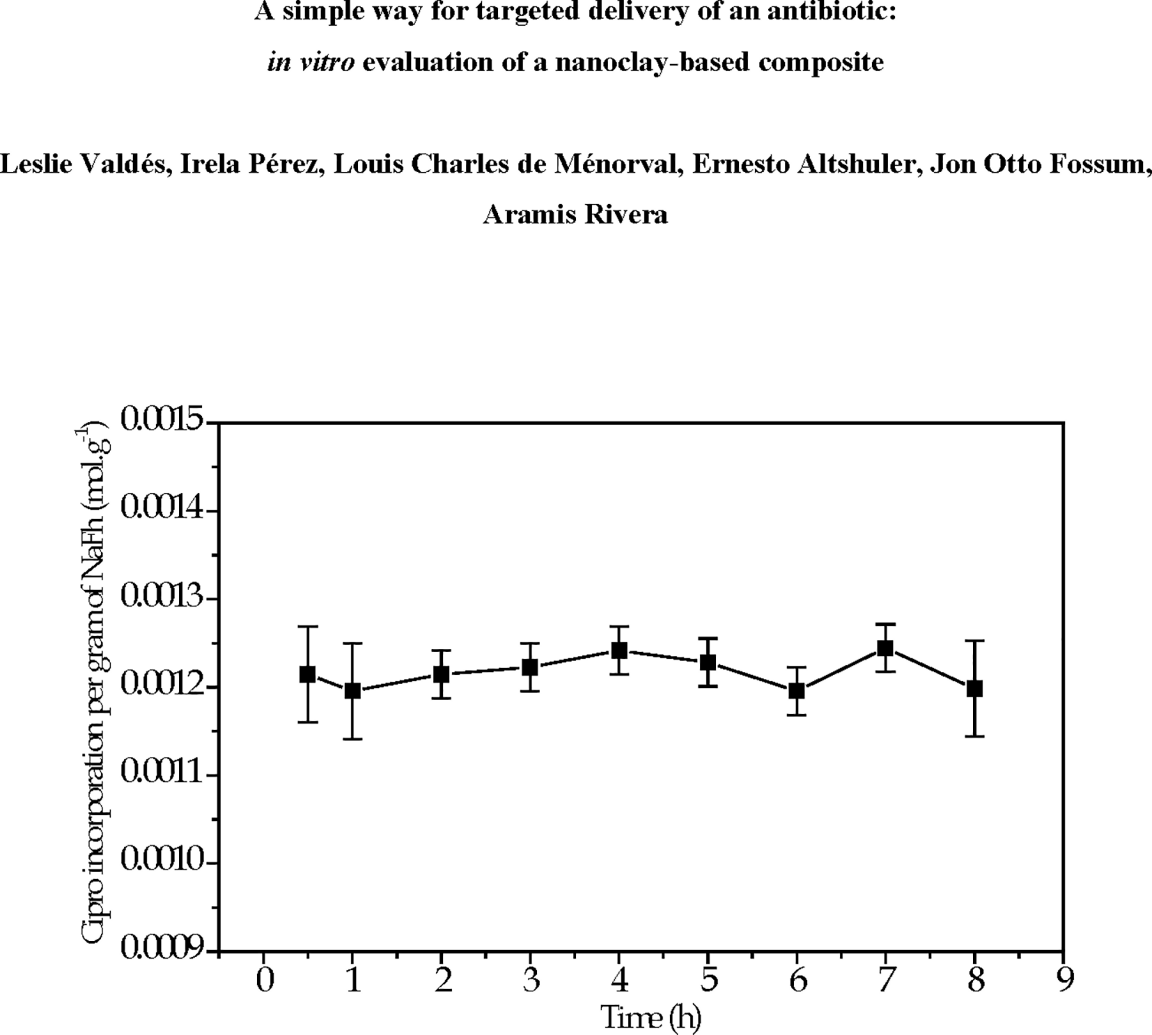
Amount of Cipro on NaFh from aqueous solution at 1.36 x 10^-2^mol L^-1^ as a function of time. The equilibration time applied was 4 h at 65 ^o^C.

Regarding the drug-clay interaction time, no relevant variations in the Cipro amount incorporated into NaFh clay ―in particular from 2 to 5 h― are observed after the interaction (see Fig 4). About 90% of Ciprofloxacin initially in solution is rapidly incorporated, remaining almost constant up to 8 h. Thus, interaction time was set to 4 h in the subsequent experiments in order to guarantee the right balance between the contact time and the appropriate Cipro incorporation. Such results indicate that the incorporation process, i.e., the amount of Cipro captured by clay takes place very quickly, which suggests a strong affinity between drug and clay. The nature of the drug-clay interaction will be discussed later on.

### Adsorption isotherm

**Fig 5:**
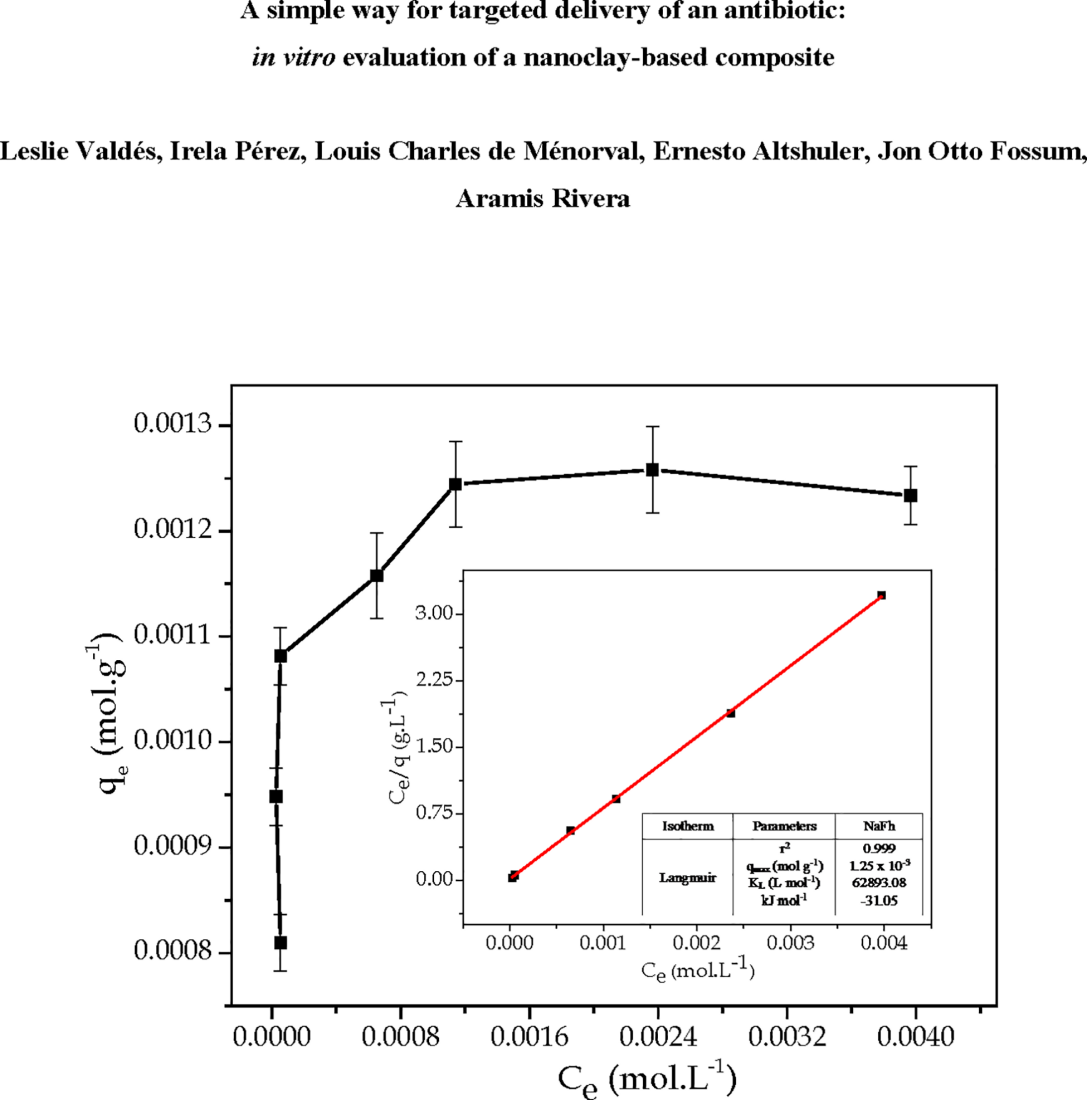
Cipro adsorption isotherms for NaFh and the parameters of adsorption isotherm models. The solid line in the inset represents Langmuir model fit.

The experimental isotherm of Cipro on NaFh at 67°C ± 3°C, optimal pH (acid) and a constant time (4 h) is shown in Fig 5. At low concentrations the drug loading is quite fast, followed by a slow approach to equilibrium until a maximum Cipro concentration is reached. It can be also observed that for the same equilibrium time, the drug loading is higher for a greater value of initial Cipro concentration. The curvature of the isotherm decreases as the Ce value increased noticeably for a small increase in q_e_.

The dependence of the adsorbent loading on the adsorbate in equilibrium at a constant temperature can be described by an isotherm equation. Langmuir, Freundlich and Dubinin–Radushkevich models were used to fit the experimental isotherm [42–44]. In the present case, the Cipro adsorption onto NaFh was best described by the Langmuir isotherm model, which is given by the following equation:

$$\frac{C_{e}}{q_{e}}=\frac{1}{q_{max}K_{L}}+\frac{1}{q_{max}}C_{e}$$

where *C*_*e*_ is the Cipro concentration in the solution at equilibrium (mol·L^-1^), *q*_*e*_ is the mass of Cipro adsorbed per unit mass of the adsorbent at equilibrium (mol·g^-1^), *q*_*max*_ is the maximum monolayer coverage capacity (mol·g^-1^) and *K*_*L*_ is the Langmuir adsorption constant (L·g^-1^) which is related to the free energy (ΔG°) of adsorption by:

$${\Delta G}^{{}^{\circ}}=-RTlnK_{L}$$

where *R* is the gas universal constant (8.31 J mol^-1^ K^-1^) and *T* is the absolute temperature (K).

The Langmuir model is based on the assumption of monolayer adsorption on a structurally homogenous adsorbent, where all sorption sites are identical and energetically equivalent. Following this model, the calculated maximum Cipro loading for the NaFh clay was 1.25 x 10^−3^ mol·g^-1^ (see inset of the Fig 5). By comparing the Cipro load determined experimentally (1.24 x 10^−3^ mol·g^-1^ ± 4.08 x 10^−5^) and that calculated by the Langmuir model, the difference is negligible. The calculated ΔG° was ‒31.05 kJ mol^-1^. The negative value of this magnitude indicates the spontaneous nature of the adsorption process, and its module suggests a physical sorption process. Similar results have been also reported by the other investigators, using smectite clays as drug support, and for the elimination pollutants [6, 45, 46].

### Characterization of the composites

Based on the results discussed in the previous sections, the best experimental conditions to obtain the composite NaFh-Cipro were the following: an equilibration time of 4 h, a Cipro initial concentration of 1.36 x 10^−2^ mol L^-1^, a temperature in the range 65–70°C, and a pH of about 4. In order to check the repeatability of the preparation method, five samples of the composite were obtained. The results indicated that the average amount of Cipro present in NaFh is 1.30 x 10^−3^ ± 1.34 x 10^−5^ mol·g^-1^ of clay. In all cases, the confidence intervals at 95%, i.e., confidence level according to the *t-student* distribution (p > 0.05), are quite narrow, indicating excellent repeatability of the method.

**Fig 6:**
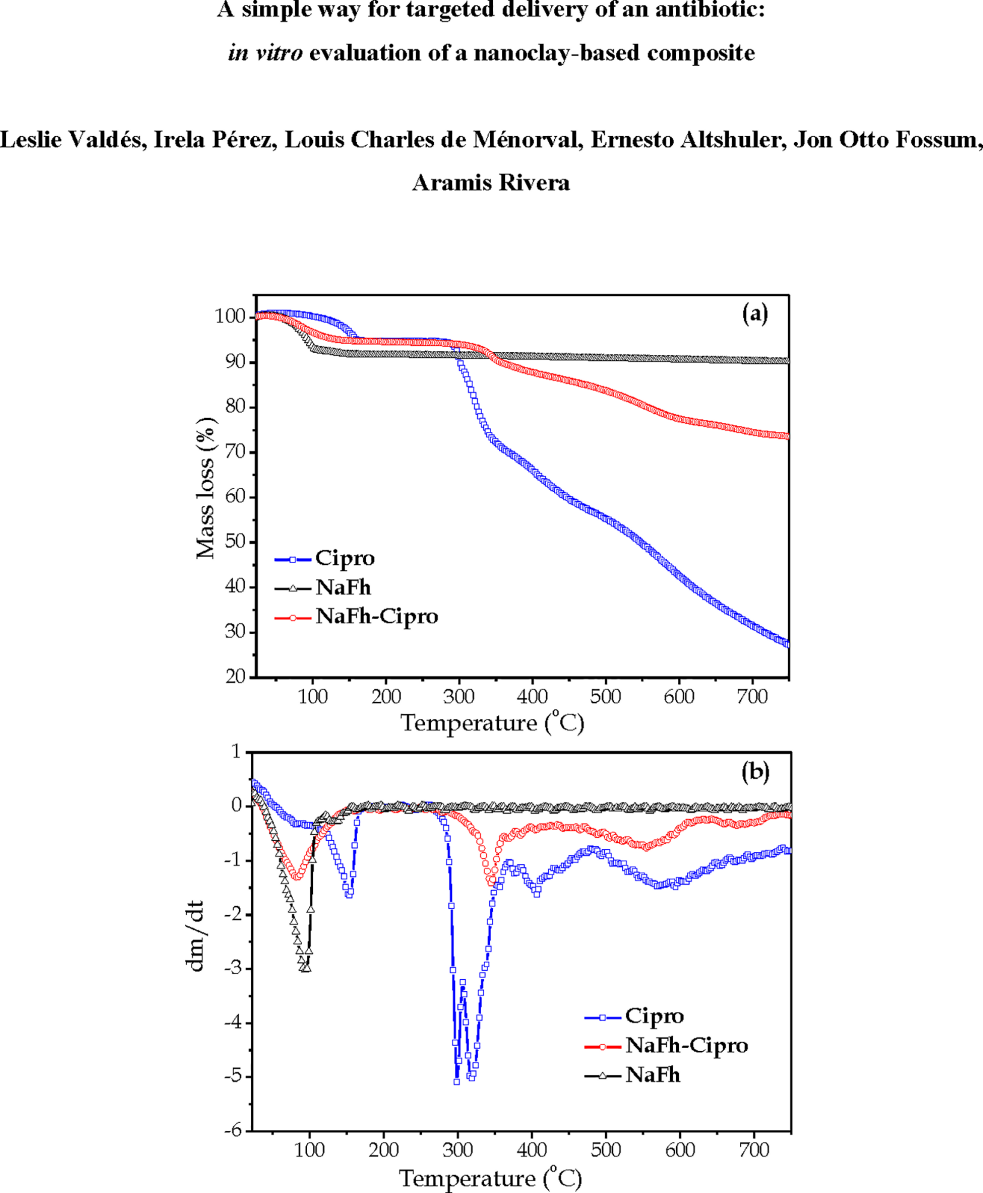
TG and differential thermogravimetry (DTG) curves–(a) and (b), respectively–for ciprofloxacin (Cipro), NaFh and the NaFh-Cipro, at 10 ^o^C/min.

The TG/DTG curves for the raw materials (Cipro and NaFh) and the composite NaFh-Cipro are shown in Fig 6. For the Cipro, five peaks of mass loss at 154, 299, 319, 406 and 571°C are identified based on previous reports [32, 47]. The Cipro decomposition takes place in three successive steps: the first one, is related to the loss of CO molecules. The second one (associated to two peaks in the DTG curve), is attributed to a loss of [C_4_H_8_N_2_H_2_ + CO]. The last step (also associated to two peaks) has been attributed to the removal of the residual drug as C_11_H_8_FNO. The TG curve for the clay indicates a mass loss until 160°C, and two peaks in the DTG curve are identified. They are attributed to desorption of mesoporous and intercalated water, around 95 ^o^C and 135 ^o^C, respectively. The dihydroxylation of NaFh occurs normally at temperatures higher than 760 ^o^C [48]. The NaFh-Cipro composite shows peaks of mass loss around 83 ^o^C and approximately at 345 ^o^C, 555 ^o^C and 678 ^o^C. The first peak is attributed to the loss of water, and the last three peaks can be ascribed to the second and third steps of the decomposition process of ciprofloxacin and the residual removal of drug, respectively, which corroborates its presence in the composite. The comparison of the DTG curves of the pure Cipro and the NaFh-Cipro composite, evidences that an increase takes place in the temperature of Cipro decomposition ―last four peaks in the Cipro thermogram― after the interaction with the clay. It indicates that the Cipro thermal stability increases in the composite, and suggests that such peaks in the composite could be assigned to Cipro molecules intercalated between the clay sheets. It also shows that the drug molecules are bonded strongly in the interlayer space of the fluorohectorite. In the composite diagram, the peak corresponding to the loss of water appears at lower temperatures and has less intensity, if compared to the pure clay. A possible explanation is as follows: in the composite, the presence of Cipro increases the interlayer space and it helps the water molecules to desorb easier. Additionally, the presence of Cipro in the clay interlayers displaces a greater number of water molecules, producing a peak of less intensity related to the pure clay.

**Fig 7:**
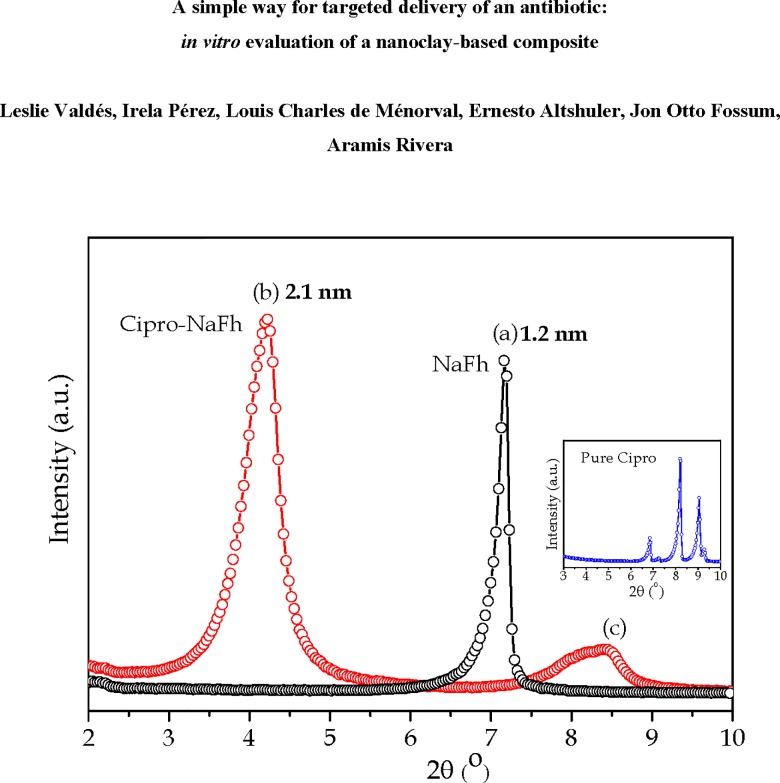
Diffraction patterns for NaFh, NaFh-Cipro composite and the pure Cipro.

Fig 7 shows the X-ray diffraction (XRD) patterns for the raw materials (Cipro and NaFh), and the NaFh-Cipro composite. In the X-ray pattern of the NaFh only one peak at 2θ = 7.2°, corresponding to the (001) Bragg reflection, is observed. This peak, marked as (a) in Fig 7 corresponds to an interlayer distance *d*_*001*_, which is close to 1.2 nm when one water layer is intercalated in the stacks in normal conditions [32, 48, 49]. The position of the Bragg peak changes when the drug is intercalated, and it appears at smaller scattering angle showing that the characteristic distance *d*_*001*_ between the clay’s crystalline sheets has increased. In the XRD pattern for the NaFh-Cipro sample two peaks are observed, which are labeled as (b) and (c) in Fig 7. Peak (b) indicates an interlayer distance along the [1] direction, *d* = 2.1 nm for the composite, in contrast to *d* = 1.2 nm, typical of the pure clay. The large dimensions of Cipro (1.20 nm x 0.74 nm) as compared to water molecules are clearly responsible for the observed increase of the interlayer distance, so we have strong evidence that the drug (probably hydrated) has been incorporated in the interlayer space of the clay. This corroborates the conclusions from the TG/DTG results. The second peak (c) is the most intense signature of the pure drug (see inset in Fig 7).

**Fig 8:**
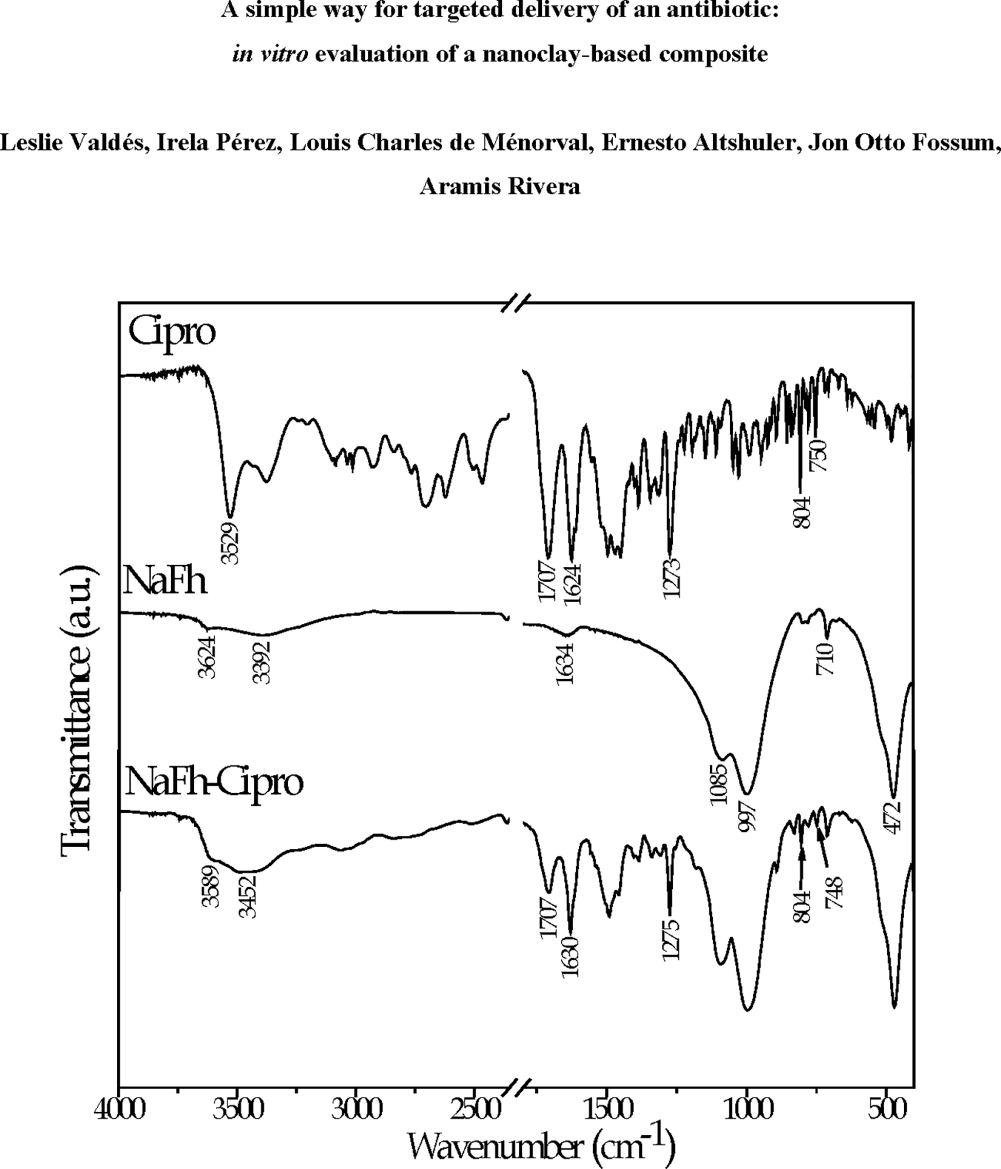
Infrared transmittance spectra for the samples ciprofloxacin (Cipro), NaFh and NaFh-Cipro. The characteristics bands are indicated for each one.

Fig 8 displays the IR transmittance spectra for the all samples (NaFh, Cipro, and NaFh-Cipro composite), indicating the wavenumbers of their characteristic bands. For the raw material NaFh, the main bands corresponding to O─H and Si─O vibrations are assigned in the spectrum [32, 50]. The main bands at 997 cm^−1^ and 472 cm^−1^ correspond to Si―O in-planes stretching and bending, respectively. The Si―O out-of-plane stretching and bending bands were also identified at 1086 cm^−1^ and 710 cm^−1^, respectively. Two discrete bands can be seen around 3500 cm^-1^, in the ─OH stretching region. The band at 3624 cm^-1^ is ascribed to OH stretching of structural hydroxyl groups. Stretching vibrations of water molecules in the interlayer space may also contribute to the ─OH bands (3392 cm^-1^). The band at 1643 cm^-1^ is also due to the presence of water, in particular to bending vibrations of H_2_O [32, 50–53].

In the Cipro spectrum, the characteristic bands appear in the region 1800–1200 cm^-1^: the bands at 1707 cm^-1^ and 1624 cm^-1^ are assigned to the C = O stretching of the carboxylic acid and of the ketone, respectively. At 1273 cm^-1^ the typical band of the COOH groups containing compounds is assigned to coupling of the carboxylic acid C**─**O stretching and O─H deformation [32, 54–57].

The composite spectrum (NaFh-Cipro) shows distinctive bands corresponding to the drug, in particular those at 1707, 1630 and 1275 cm^-1^, which confirms the presence of Cipro in the clay (see Fig 8). In this respect, no significant variations in the wavenumbers of the first and last band assigned to Cipro are observed. However, it is possible to identify a shift of the Cipro band from 1624 cm^-1^ to 1630 cm^-1^ ―corresponding to C = O stretching of ketone― after the interaction with the clay. Such shift in the NaFh-Cipro spectrum in comparison with that of Cipro alone, suggests the existence of interaction between the drug ketone group and the clay. Such result has been previously reported for a similar system before modification of the clay [32]. The bands in the low frequency region at 804 cm^-1^ and 748 cm^-1^ do not show significant variations relative to the pure drug spectrum.

A further interaction between Cipro and clay is detected, as follows. In the spectrum of the NaFh-Cipro composite, two shallow bands around 3589 cm^−1^ and 3452 cm^−1^ are identified. The incorporation of Cipro in the interlayer space could modify the environment around the structural hydroxyl groups and the water, which would imply modification of the adsorption bands assigned to these groups in the clay. Such interaction could also affect the vibrational normal modes of the organic molecule, resulting in modification of the band assigned to pure Cipro (with center near 3529 cm^-1^, corresponding to the O─H stretching vibration) due to interactions via hydrogen bonding between the Cipro positively charged groups and the hydroxyl groups of the clay, as well as with the water present in the interlayer space.

**Fig 9:**
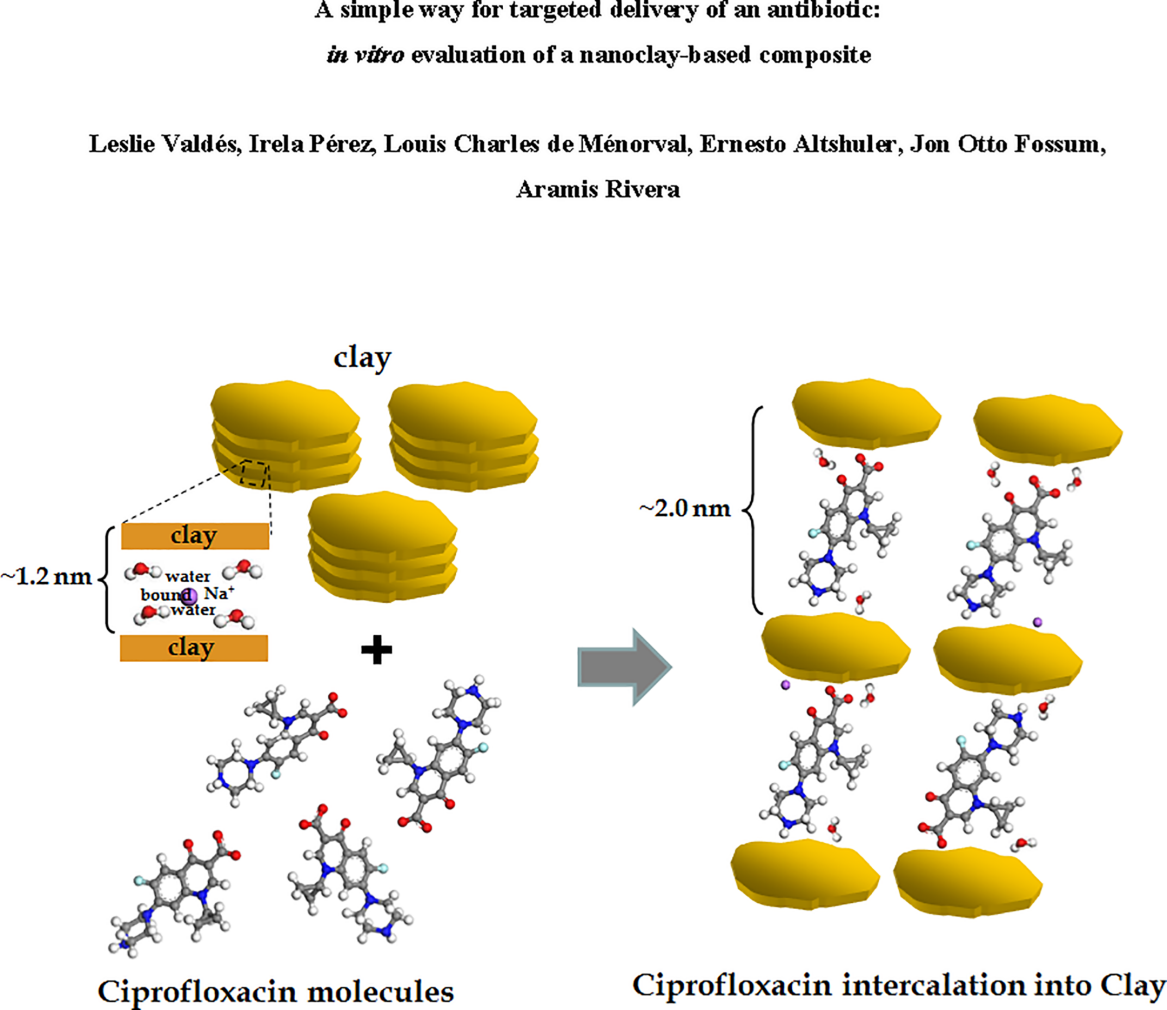
Sketch of the incorporation of Cipro into the NaFh clay. Left panel: separate clay and Cipro in solution. Right panel: NaFh clay with Cipro molecules intercalated in the interlayer space.

Based on the basal spacings of the (001) planes and the molecular size of the intercalated species, a schematic representation of the Cipro incorporation into NaFh is sketched in Fig 9.

### In vitro drug release

**Fig 10:**
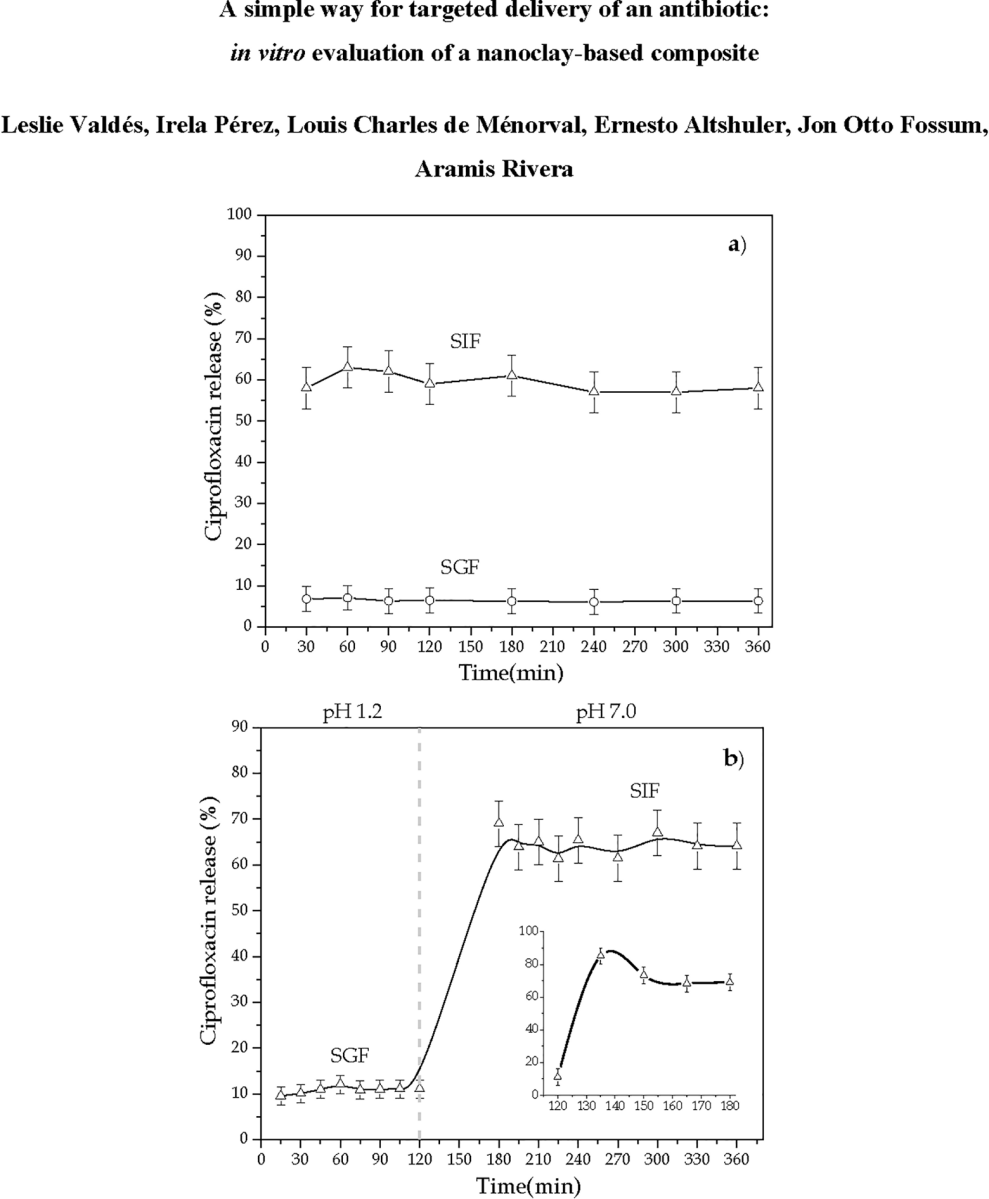
*In vitro* release profiles of Cipro from composite (equivalents to 2.53 x 10^−5^ mol of drug/0.3g of NaFh-Cipro): (a) simulated gastric fluid (SGF, pH = 1.2) and simulated intestinal fluid (SIF, pH = 7.0) at 37°C; (b) continuous release profiles from NaFh-Cipro (n = 3). The lines in (b) are a guide to the axis.

The release profiles of Ciprofloxacin from the NaFh-Cipro composite at 37 ^o^C over 6 h are shown in Fig 10. They were obtained from *in vitro* release experiments performed on the best composite obtained. As can be seen in the figure, the drug release is strongly dependent on pH. Fig 10 (A) displays two separate release experiments performed at pH values of 1.2 and 7.0, i.e., those corresponding to the environments of the stomach and the large intestine, respectively. In both cases, most of the release takes place at times shorter than 30 minutes, then reaching stable plateaus near 5% and 60% of drug delivery for stomach and intestine conditions, respectively. So, *the drug release at the intestinal level is approximately ten times bigger than that in the stomach*: our composite manages to deliver a significant proportion of its cargo at the desired place. Moreover, the composite obtained is able to retain the drug for a substantially larger time in comparison with commercial forms existing in the market. Typically, the release profile of commercial Cipro formulations show that around 80–100% of the drug is released within a period of 10–30 min [1, 58, 59]. Fig 10 (B) shows a “continuous delivery assay” where the pH of the solution is switched from the stomach to the intestine environments as time goes by: the drug release values are consistent with those corresponding to the separate experiments shown in Fig 10 (A). The only feature that is worth pointing out in the continuous assay, is a moderate release peak (shown in the inset of the figure) that occurs shortly after the pH change.

To evaluate the release mechanism Cipro from the composite, it is necessary to take into account different aspects: interactions drug-clay in the composite, clay structural characteristics, functional groups and physical-chemical properties of the drug, as well as the chemical species from the simulated fluids. For example, in SGF the protonated Cipro is clearly retained into the clay resulting in very slow release of the drug, which suggests a high affinity of Cipro for the clay, whereas in SIF the release is faster. If we assume that (1) Cipro release follows an ionic exchange process between Cipro molecules into interlayer space and cations from the liquid medium (Na^+^ and H^+^ in the SGJ), (2) the compensating cations remaining in the clay under study are basically sodium, and (3) Cipro incorporation takes place at acid pH (i.e., Cipro is in its protonated form); it is then expected that exchange of both species―Cipro from clay by sodium or protons from the liquid medium― it is not so favoured.

However, in SIF where the species in the dissolution medium are basically K^+^ and Na^+^, the Cipro ―that initially is in its protonated form in the composite― begins to loose protons prevailing its zwitterionic form, which results in a sizable release of the drug. It suggests that the Cipro in its dipole form is less affine to the clay. In addition, the presence in the dissolution medium of a different interchangeable cation (like potassium) from the clay native sodium cation facilitates the leaving of Cipro from the composite. Such facts suggest that the Cipro release from NaFh-Cipro composite is controlled by more than one mechanism. The “pure” diffusion of the Cipro, and/or the ionic exchange of species depending on the nature of the dissolution media.

### Conclusions

We have proposed a new composite obtained by the intercalation of Ciprofloxacin into the synthetic clay Na-fluorohectorite aimed at the controlled release of the drug at the large intestine, which minimizes the side effects associated to burst release in the stomach, while eluding coating technology.

A systematic physical-chemical study revealed that the best composite is obtained by clay-drug contact in a liquid medium at acid pH, with a drug concentration of 1.36 x 10^−2^ mol L^-1^, 65 ^o^C, and 4 hours of contact. In such conditions, the clay loads as much as 1.3 x 10^−3^ mol of Ciprofloxacin per gram of material. X-ray diffraction and other analytical techniques reveal that the drug molecules are intercalated between the clay layers.

In vitro release assays demonstrated that less than 10% of the composite cargo is released in a simulated gastric fluid (pH = 1.2), while more than 60% of the Ciprofloxacin is release in a simulated intestinal fluid (pH = 7.0). The same behavior is observed when the composite is in a medium than changes its pH from 1.2 to 7.0, demonstrating its ability to release most of the drug at the desired target, i.e., the large intestine.

These results proved the potential of the new nano-material as a low-cost alternative to deliver a wide-spectrum antibiotic in a desired location of the gastrointestinal tract, avoiding the use of coating technology and minimizing undesirable side-effects.
